# High cardiovascular risks in a North Indian Agarwal community: a case series

**DOI:** 10.1186/1757-1626-2-7870

**Published:** 2009-06-03

**Authors:** Rajeev Gupta, Mukta Agrawal

**Affiliations:** 1 Department of MedicineFortis Escorts Hospital, Jaipur 302017India; 2 Department of MedicineMahatma Gandhi Medical College, Jaipur 302022India; 3 Department of Home ScienceUniversity of Rajasthan, Jaipur 302004India

## Abstract

Community specific cardiovascular disease risk factor studies may be useful to evaluate genetic versus environmental causes. Agarwal is a high profile business community in India. A family based narrative from this community reveals a high prevalence of cardiovascular diseases- stroke and coronary heart disease. This is associated with high prevalence of multiple cardiovascular risk factors such as central obesity, hypertension, lipid abnormalities and diabetes. Another study in the same community in Jaipur reveals high prevalence of obesity associated with low physical activity and high dietary calorie and fat intake. Lifestyle factors appear to be mediator of the cardiovascular epidemic in this community.

## Introduction

Cardiovascular diseases are major problems in India [1]. Epidemiological studies have shown that coronary heart disease in India is mediated by a handful of well known risk factors, such as, abnormal lipids (low apolipoprotein A, high apolipoprotein B), hypertension, diabetes, obesity, smoking, psychosocial stress, diet low in fruits and vegetables, and sedentary lifestyle [2]. However, in many individuals with coronary heart disease, especially that occurring before the age of 55 years, genetic factors or gene-environment interactions could be important [3]. Genetic factors have been extensively studied in recent years and multiple locations on chromosomes as well as single nucleotide polymorphisms have been identified [4]. However, after the development of whole genome analysis techniques it has been concluded that the initial reports of genetic polymorphisms were false positive and barring a few important genes that have been mechanistically linked with pathophysiology of atherosclerosis, most of the polymorphisms could not be replicated on large scale analyses [5]. Are there specific families or family groups that are important in this regard is unknown and it has been suggested that family based studies and narratives could be important.

Serendipitously, it has been observed that among one of the business communities of north India, the Agarwal community, there is a high prevalence of various types of cardiovascular disease, especially coronary heart disease. To highlight importance of lifestyle and/or genetic influences as suggested by a family pedigree based analysis we report a narrative from a typical family belonging to this ethnic group. This clan has 10-15 million individuals that are spread worldwide and are akin to business communities elsewhere in the world [6]. It is said that the initial forebear of this clan was in the north Indian state of Haryana about 100 miles west of Delhi and lived about 1000 years BC [7]. He had seventeen sons from different queens, who were all sisters, and the clan has been subdivided into these subgroups accordingly. Even to this date most of the subjects in this community marry among themselves only and there is a restriction of inter-marriages within one of the groups. This clan has a high profile presence among business and professional strata in India and a list of top 40 richest Indians by Forbes Inc. included 18 individuals from this clan [8]. Detailed anthropological and sociological characteristics of this group have not been reported.

## Case presentations

I belong to the clan of Agarwals. Our ancient village is close to Delhi in Haryana, from where most of the Agarwals originate, and the family members have migrated far and wide [6]. Very early in childhood I learnt that my paternal grandmother (1892-1951) had severe hypertension and developed stroke in early 1950's. She died within 6 months with complications of prolonged immobilization and urinary infection at age of 60 years. My paternal grandfather (1885-1971) was as overseer and took premature retirement from government service at age of 50 years after it was known that he had high blood pressure (BP) that was uncontrolled with the then available medicines (reserpine, hydrallazine, diuretics). By this time he had sired multiple children. 4 sons (born 1912, 1920, 1922, 1925) and 2 daughters (born 1914, 1919) lived into adulthood. Papa grandfather lived to the ripe age of 85 and died in 1971. In the last 10 years of life he developed multiple strokes, recovered from 2 of them with almost no sequelae but the third left him paralysed and aphasic. He survived in this state for more than 2 years before debility and chest infection took him away.

My eldest uncle (1912-1986) was a physician, a heavy smoker and liked good Indian food full of sweets and fats. He was also very active physically. At age 45 he developed hypertension which he tried to control by giving up smoking and diet restriction but by age of 50 years he developed severe angina, survived one episode of myocardial infarction, continued to have disabling angina, developed severe peripheral vascular disease and intermittent claudication and was managed on drugs with severe restriction of activities. At the age of 74 years, he developed another myocardial infarction, had a cardioembolic stroke and passed away. All three of his sons and one of two daughters have high blood pressure. The eldest son (b 1936) was user of chewed tobacco. He developed angina at age of 45 years, was on medication for a long time but at age of 56 years underwent triple coronary bypass surgery. One daughter (1936-1984) developed end stage renal failure and hypertension and passed away after prolonged dialysis treatment. The other two sons (b 1942 and 1944) are hypertensive on regular treatment and the youngest child a daughter (b 1948) is alright. There are nine fourth generation children in this arm of the family, eight are more than 35 years and there is no evidence of hypertension or diabetes in any of them. Two of these children are obese using current definitions.

The eldest aunt (1914-1987) had severe hypertension from age of 40 years and was on a variety of medications off and on. She was diagnosed with diabetes at age 60 years but was very irregular on diet and almost no physical activity. She developed congestive heart failure at age 64 years, had an episode of thrombotic stroke (by this time neuro-imaging had arrived) at age 65 years, recovered significantly but had another stroke at age 71 years and was confined to bed for 2 years before death. All her three daughters and a son have hypertension, all are on multiple drugs including beta blockers, ACE inhibitors and calcium channel blockers. There is clinical and echocardiographic evidence of left ventricular hypertrophy in all and evidence of early diastolic heart failure is present in all the daughters. A large number of fourth generation children (n = 14) exist in this family. Three of these have high blood pressure and there is no diabetes. One boy (b 1951) developed acute myocardial infarction at 57 years of age and underwent coronary angioplasty. The second aunt (1919-1992) developed hypertension and angina at age of 55 years, survived this with lots of physical activity and economic hardships. At age 73 years died suddenly of cardiac arrest. Retrospective history revealed possible acute coronary event. Her four sons and one daughter all have high BP, three have diabetes in addition and all are on regular medical treatment. The eldest son (1936-2008) died of acute myocardial infarction at age 72 years. A younger son (b 1951) developed angina at age 50 years, coronary angiography revealed small vessel disease and is on regular medical treatment.

The second uncle (1920-1995), was a railway officer. He developed hypertension at young age of 45 years and was on treatment for a long time. He had angina and congestive heart failure, developed stroke with severe disability and aphasia at age 70 years, developed diabetes, survived for 5 years and passed away at age 75 years. His children, two sons and two daughters all have high blood pressure and are on regular treatment. The third uncle (1922-2004) was a physician. He had an episode of acute myocardial infarction at age 57 years soon after retirement from government service. Had another infarction at age 60 years and underwent quadruple coronary artery bypass surgery in 1983. He also had hypertension, and reduced left ventricular function and continued for a very long time on the then popular evidence based therapies (beta blockers, aspirin, angiotensin receptor blockers and calcium channel blockers). He had progressive congestive heart failure, sick sinus syndrome requiring dual chamber pacing, developed chronic renal failure and passed away at age 83 years of complications of the latter conditions. His elder son a physician (b 1952), developed acute myocardial infarction at age 49 years, survived the acute episode, coronary angioplasty at multiple sites was performed and is on regular medications. The other son and daughter have high blood pressure and have borderline high cholesterol (~200 mg/dl) and are on regular treatment. The fourth uncle (1925-2007) was a heavy smoker and a petty businessman in the native village. He was normotensive and non-diabetic but at age of 82 years developed severe chest pain and died within 4 hour of symptom onset. His two sons and one of his two daughters have high BP and are on regular medication.

## Risk factor profile

Table 1 shows prevalence of major cardiovascular risk factors in successive generations and their spouses in the present family. Smoking or tobacco use is very low and across generations has remained so. Combining the smoking prevalence for the total cohort shows a prevalence of 5.6% which is much below the national average [1]. A high prevalence of central obesity (waist size > 90 cm) is observed and appears to be increasing in the younger generations. A very high prevalence of hypertension in the first three generations that have reached the middle-age is noted. A significant prevalence of diabetes is observed. However, prevalence of these two risk factors are similar to the national data [1]. Increasing use of cholesterol lowering treatment reflects current clinical practice, high educational status of family members, and influence of social networking in a family cohort [9]. An important observation is high incidence of coronary artery disease in the second and third generations which is more than previously reported studies from India [1]. The incidence of cerebrovascular disease is also high but seems to be levelling off in later generations. There is no reported case of cancer in these generations either in the direct descendents or the spouses.

**Table 1.** Prevalence of major cardiovascular risk factors and cardiovascular disease in various generations

	Number	Smoking	Central obesity	High blood pressure	Diabetes	High Cholesterol on Treatment	Coronary artery disease	Cerebrovascular disease
First generation	2	0	0	2 (100.0)	0	0	0 (0.0)	2 (100.0)
Second generation	6	2 (33.3)	2 (33.3)	6 (100.0)	2 (33.3)	2 (33.3)	4 (66.7)	2 (33.3)
Second generation spouse	6	0	3 (50.0)	3 (50.0)	1 (16.7)	1 (16.7)	3 (50.0)	0
Third generation	25	0	13 (52.0)	19 (76.0)	4 (14.8)	13 (48.1)	4 (16.0)	1 (4.0)
Third generation spouse	20	0	12 (60.0)	5 (25.0)	1 (5.0)	1 (5.0)	1 (5.0)	0
Fourth generation >35 yr	18/50	0	11 (61.1)	9 (50.0)	0	2 (11.1)	1 (5.6)	0
Fourth generation spouse >35 yr	12/25	3 (25.0)	8 (66.7)	3 (25.0)	1 (8.9)	0	0	0
Total	89/134	5 (5.6)	59 (66.3)	47 (52.8)	9 (10.1)	16 (18.0)	13 (14.6)	5 (5.6)

The questions that emanate from this cohort study are (i) is the family genetically susceptible to hypertension and other cardiovascular risk factors; (ii) does the high incidence of atherosclerotic vascular disease reflect genetic influence; (iii) are gene-environment interactions to blame; (iv) are the primordial cardiovascular risk factors (diet, sedentary lifestyle, etc) important; or (v) do the changes in risk factors in successive generations reflect the epidemiological and societal evolution in India.

## Cardiovascular risk factors

A large number of factors of risk for cardiovascular diseases have been identified [3]. The medical statistician Sir Austin Bradford-Hill summarised the criteria used to assess significance of associations between a disease and a supposed causative agent [10]. The criteria for causality include strength of association, consistency, specificity, temporality, biological gradient, biological plausibility, biological coherence, experimental evidence and analogy. The traditional risk factors that fulfil most of these criteria are high total and LDL cholesterol, high blood pressure, low HDL cholesterol, diabetes, smoking, age and male sex [3]. All these risk factors are important proximate determinants of cardiovascular diseases and the effects are cumulative as well as multiplicative. The present family has a high prevalence of many of these risk factors (Table 1) and it is unsurprising that the incidence of cardiovascular disease is high. The INTERHEART study has reported that these standard risk factors explain more than 90% of incident acute myocardial infarctions [2]. In the same study, genetic analyses of more than 1400 single nucleotide polymorphisms revealed that genes explain only about 2% of the risk beyond the standard risk factors [11].

The natural history of atherosclerotic disease also involves interaction of multiple risk factors that include social and economic factors, genetic factors, primordial risk factors, and traditional risk factors [3]. There is a strong influence of environmental factors such as diet, exercise, adiposity and medications at every level of risk factor and disease progression [3]. In the index Agarwal family there is high prevalence of coronary heart disease (14.6%) that is almost twice than the population based studies in Jaipur (7.6%) [12]. This correlates well with a high prevalence of multiple cardiovascular risk in this group which is significantly greater than risk factors reported in contemporary population based studies at Jaipur (Table 2) [12,13]. The prevalence of risk factors in this cohort is also more than in a high risk group of north Indian Punjabi Bhatia community studied in Jaipur [14]. It is observed that prevalence of central obesity (surrogate for the metabolic syndrome), hypertension and hypercholesterolemia is greater in this community while prevalence of diabetes is similar.

**Table 2.** Prevalence (%) of Multiple Cardiovascular Risk Factors in the Study Cohort Compared with Contemporary Studies in Jaipur

	Population based study in Jaipur (JHW-2)^13^	High risk population group, e.g., Bhatia (JHW-3)^14^	The present Agarwal family
	**N = 955**	**N = 421**	**N = 89**
Smoking/tobacco use	21.9	14.4	5.6
Truncal obesity	40.9	46.6	66.3
Hypertension	27.5	38.9	52.8
Hypercholesterolemia	35.7 (<1% on treatment)	31.1 (<1% on treatment)	18.0 on treatment
Diabetes	7.9	8.6	10.1
Metabolic syndrome	24.3	32.7	-
Coronary heart disease	7.6	-	14.6

There is a strong interaction of risk factors such as low socioeconomic status, socioeconomic upsurge, childhood nutrition, childhood growth pattern, educational level, job stress and many others with development of cardiovascular risk factors in childhood and adulthood and subsequent incidence of cardiovascular diseases [15]. Immediate primordial risk factors for cardiovascular diseases include smoking or tobacco use, physical inactivity, and dietary indiscretion [3]. We have closely observed the social evolution of the present family and have noted that the generational roots of the family in late nineteenth century were rural lower middle class. Most of the second generation children were born underweight, remained undernourished in childhood and young adulthood and it was only after migration of these members to either near or distant urban locations the affluence increased. Hypertension developed early. Lipid levels were not measured. Unsurprisingly, cardiovascular diseases, especially, coronary heart disease developed prematurely in all the second generation individuals, although all lived to a long age. The third and subsequent generations of the family has so far shown a lower prevalence of multiple risk factors as well as manifest cardiovascular diseases. Whether it is due to genetic drift, epigenetic factors, change in early life events or improving socioeconomic environments is not clear [16]. More studies of gene-environmental interactions among this and other high-risk groups are needed.

Data on prevalence of specific cardiovascular risk factors among the Agarwal community are not available. A pilot study of diet and physical activity among Agarwal women in Jaipur reported high consumption of calories and fats and a low physical activity level [17]. In this study of 137 middle aged Agarwal subjects (men 81, women 56) a high prevalence of obesity was seen (Table 3). It was also observed that consumption of calories (mean 2487 ± 635 calories/day) and fats (89.4 ± 26.0 g/day) was high and physical activity levels were low in both men and women. It was also observed that during fasting and feasting days (more than 4 times a month) the consumption of calories was > 4000 calories/day [17]. Similar situation may be prevalent in all the middle and upper-middle socioeconomic status members of the community although more studies that include larger and representative samples are required.

**Table 3.** Calorie and fat intake, physical activity levels and prevalence of obesity and other risk factors in a pilot study among Agrawal community in Jaipur

	Men (n = 81)	Women (n = 56)	Total (n = 137)
Body mass index (kg/m^2^)	25.5 ± 3.9	24.8 ± 5.0	25.2 ± 4.4
Waist size (cm)	94.1 ± 10.8	85.4 ± 11.6	90.6 ± 11.9
Obese (BMI >25 kg/m^2^)	49 (60.5)	27 (48.2)	76 (55.5)
Vigorous physical activity (min/day)	18.4 ± 33.5	35.2 ± 55.8	26.8 ± 44.7
Calories /day	2790 ± 702	2296 ± 508	2487 ± 635
Proteins g/day	75.8 ± 22.2	59.8 ± 15.3	65.9 ± 19.7
Fat g/day	96.8 ± 25.6	85.7 ± 25.5	89.4 ± 26.0
Visible fats g/day	62.3 ± 18.2	53.9 ± 33.0	57.2 ± 15.1
Fat energy %/day	31.2	33.2	
Green leafy vegetables g/day	38.9 ± 17.5	41.0 ± 15.6	40.2 ± 16.3
Fruits g/day	548.8 ± 351.9	480.5 ± 177.1	506.9 ± 258.3

## Conclusions

Can the epidemic of cardiovascular diseases in a community be reversed? Definitely yes [18]. Focus on social determinants of health and improving socioeconomic status, as exemplified by increasing educational status, in a community leads to better maternal nutrition, lower incidence of low birth-weight children, lesser childhood and adolescent obesity and lower smoking behaviours [19]. It ultimately leads to better adulthood nutrition, lower incidence of obesity and multiple metabolic cardiovascular risk factors and to decreasing incidence of stroke and coronary heart disease [20]. This has been reported from multiple Western European and North American populations [20] and is urgently needed among high risk subgroups, such as Agarwal community, in India and other low and middle income countries.

## List of abbreviations

HDL: High density lipoprotein
LDL: Low density lipoprotein
